# Id1 induces apoptosis through inhibition of RORgammat expression

**DOI:** 10.1186/1471-2172-9-20

**Published:** 2008-05-19

**Authors:** Yuanzheng Yang, Hong-Cheng Wang, Xiao-Hong Sun

**Affiliations:** 1 Immunobiology and Cancer Program, Oklahoma Medical Research Foundation, 825 NE 13th St, Oklahoma City, OK 73104, USA

## Abstract

**Background:**

Basic helix-loop-helix E proteins are transcription factors that play crucial roles in T cell development by controlling thymocyte proliferation, differentiation and survival. E protein functions can be repressed by their naturally occurring inhibitors, Id proteins (Id1-4). Transgenic expression of Id1 blocks T cell development and causes massive apoptosis of developing thymocytes. However, the underlying mechanisms are not entirely understood due to relatively little knowledge of the target genes regulated by E proteins.

**Results:**

We designed a unique strategy to search for genes directly controlled by E proteins and found RORγt to be a top candidate. Using microarray analyses and reverse-transcriptase PCR assays, we showed that Id1 expression diminished RORγt mRNA levels in T cell lines and primary thymocytes while induction of E protein activity restored RORγt expression. E proteins were found to specifically bind to the promoter region of RORγt, suggesting their role in activating transcription of the gene. Functional significance of E protein-controlled RORγt expression was established based on the finding that RORγt rescued apoptosis caused by Id1 overexpression. Furthermore, expression of RORγt prevented Id1-induced p38 MAP kinase hyper-activation.

**Conclusion:**

These results suggest that E protein-dependent RORγt gene expression aids the survival of developing thymocytes, which provides a possible explanation for the massive apoptosis found in Id1 transgenic mice.

## Background

T lymphocytes differentiate in the thymus from multipotent progenitors derived from the bone marrow. The developmental program can be monitored by expression of CD4 and CD8 surface markers, and progenitors advance sequentially through the double negative (DN), double positive (DP) and CD4 or CD8 single positive (SP) stages [1,2]. Within the DN stage, development can be sub-divided into 4 phases based on CD44 and CD25 expression [3,4]. Elaborate selection schemes ensure each developing thymocyte expresses a functional T cell receptor with appropriate avidity to foreign peptides presented by self MHC while simultaneously maintaining tolerance to self-antigens [5,6]. These selection processes involve precise regulation of cell proliferation and apoptosis. Ultimately, mechanisms are in place to ensure desirable mature thymocytes survive and exit the thymus.

T cell development is largely driven by the function of various transcription factors and signaling events originating from cytokine and T cell receptors. One group of transcription factors involved in this process is the basic helix-loop-helix (bHLH) family of proteins. E47 and E12 (each encoded by the E2A gene), as well as HEB, are the predominant bHLH proteins expressed in T cells, which are collectively called E proteins [7,8]. Disruption of either the E2A or HEB gene results in partial impairment of T cell development at several critical checkpoints, such as selection processes involving pre-TCR and TCR [9-12]. The function of E proteins can be blocked by their natural inhibitors, Id proteins [13,14]. While the Id3 gene is turned on following pre-TCR and TCR signaling [15,16], the Id1 gene has recently been found to be specifically activated during T cell negative selection [17], a process that eliminates self-reacting T cells. This finding suggests a physiological role for Id1 in triggering T cell death. Consistently, T cell specific overexpression of Id1 in transgenic mice diminishes the function of both E2A and HEB proteins and results in a severe developmental arrest at the earliest progenitor stages that is also accompanied by massive apoptosis [18-20]. Despite the dramatic phenotypic changes resulting from E protein deficiency, the underlying molecular mechanisms remain poorly understood. Current knowledge about genes directly controlled by E proteins cannot satisfactorily explain the phenotypes of various mutant mouse strains defective in E protein function. For example, diminished expression of pre-T cell receptor α or impaired TCR rearrangement in the absence of E protein function would have resulted in developmental arrests at the DN3 stage instead of the DN1 to DN2 transition seen in Id1 transgenic or E2A deficient mice [21,22]. Therefore, intense efforts have been devoted to identifying new targets of E proteins. However, it has been a challenge to distinguish between those genes directly controlled by E proteins and those that reflect secondary changes in E protein activities. Here, we report a strategy that is designed to increase the probability of obtaining direct targets of E proteins using microarray analyses.

One of the best candidates we obtained using this strategy was the gene encoding RORγ transcription factors. In T lineage cells, an alternative form, RORγt, is expressed from an internal promoter and is believed to play a role in the survival of double positive thymocytes [23]. RORγ deficient mice exhibit reduced thymic cellularity and excessive apoptosis, which is reminiscent of the phenotype of Id1 transgenic mice [24,25]. RORγt is also thought to inhibit the proliferation of DP thymocytes, which may be part of an integral mechanism balancing thymocyte proliferation and survival following pre-TCR signaling [26]. Interestingly, E protein function is also regulated by pre-TCR signaling through expression of the Id3 gene, which is driven by Egr transcription factors [15]. Therefore, it has been proposed that the interplay between RORγt, Egr3 and E proteins plays an important role at the transition from DN to DP stages [26]. We have shown here that inhibition of E protein function by Id1 leads to apoptosis in a DP lymphoma cell line, and co-expression of Id1 with RORγt rescued cell death in these cells. These results provide a possible explanation for the massive apoptosis occurring in DP thymocytes in Id1 transgenic mice, namely, reduced RORγt expression and diminished thymocyte survival due to inhibition of E protein function.

## Results

### Alterations in gene expression by loss or gain of E protein function

To understand the molecular mechanisms by which E proteins control T cell development, we sought for genes whose expression is directly regulated by E2A transcription factors using DNA microarray analyses. Our past attempts to compare gene expression profiles in defined thymocyte subsets isolated from wild-type and Id1 transgenic mice were not fruitful due to indirect influences on gene expression created by the abnormal cellular environment in which Id1 transgenic thymocytes develop. For example, high levels of TNFα and IFNγ in Id1 transgenic thymuses resulted in changes in genes induced by these cytokines even though E2A is unlikely to directly regulate these genes (Yang and Sun, data not shown) [27]. Therefore, we made use of a CD4 and CD8 double positive T lymphoma cell line, 16610D9, which expresses high levels of E2A proteins.

**Figure 1:**
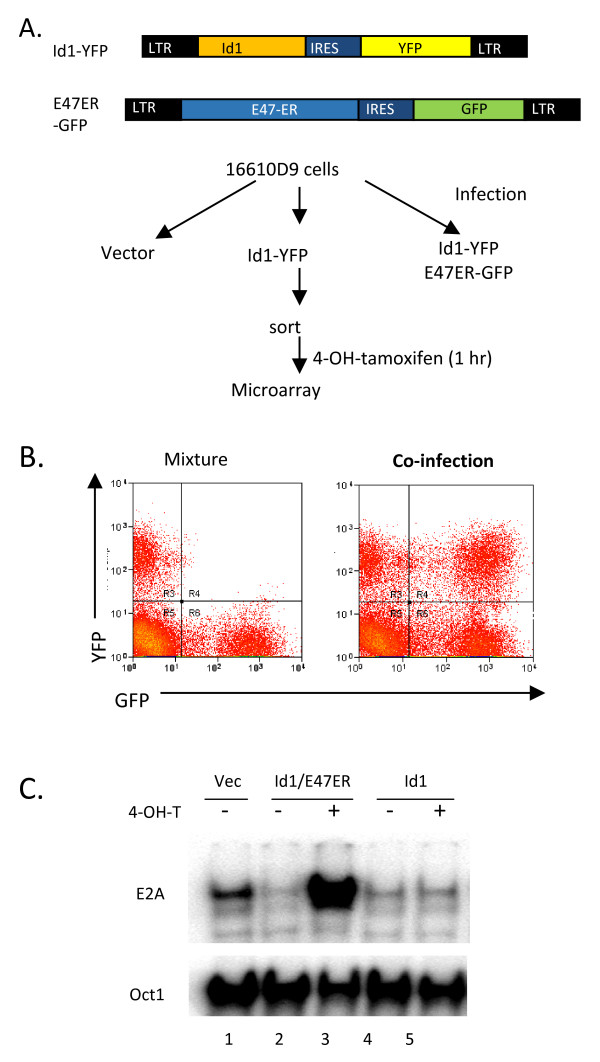
**Alteration of E2A activity in 16610D9 cells**. (A) Scheme of retroviral infection and treatment of cells used for microarray analyses. Retroviral constructs are diagrammed with key components labeled. (B) FACS analysis of 16610D9 cells infected with YFP and GFP expressing retroviruses, individually or together. The left panel shows the profile of a mixture of cells infected with either YFP or GFP expressing viruses. The right panel represents the profile of cells co-infected with both types of viruses. (C) Electrophoretic mobility shift assays of E2A DNA binding activity in indicated cells with (+) or without (-) 4-OHT treatment. Oct1 binding activities were used as controls for the amount and quality of nuclear extracts. Experimental procedures are as described [19].

To increase the probability of identifying direct targets of E2A, we designed a strategy to single out genes whose expression is inhibited by Id1 and then turned back on immediately after inducible E2A expression (Fig. 1). In this experiment, 16610D9 cells were transduced with retroviruses expressing Id1 or E47-ER proteins individually, or together. E47-ER is a fusion protein between the full-length E47 and a modified hormone-binding domain of human estrogen receptor, which has selectively higher affinity to tamoxifen than estrogen [28]. Induction with tamoxifen releases E47-ER from the cytoplasm to the nucleus and allows it to bind DNA. YFP and GFP were also expressed from an internal ribosome entry site along with Id1 and E47-ER, respectively. As negative controls, cells were transduced with empty vectors that expressed only GFP. To ensure a clear separation of cells expressing GFP or YFP, as well as both, we conducted control experiments to establish sorting conditions. As shown in Fig. 1B, cells individually transduced with YFP and GFP expressing vector control viruses were clearly separated when they were mixed. In contrast, when cells were co-infected with both types of viruses, three populations which were single or double positive for YFP or GFP, were easily distinguishable. Transduced cells were then sorted based on fluorescence from GFP and YFP. These cells were treated with 4-hydroxyl-tamoxifen (4-OHT) or vehicle control for 1 hour. Cells were then harvested for isolation of total RNA to be used in microarray studies and for preparation of nuclear extracts. Electrophoretic mobility shift assays shown in Fig. 1C demonstrated that the E-box binding activity of E proteins was inhibited by expression of Id1 in the absence or presence of 4-OHT (lane1 versus 4 and 5). In cells transduced with both Id1 and E47-ER expressing retroviruses, E box binding activities were reduced in cells without 4-OHT treatment but markedly increased upon induction of E47-ER with 4-OHT (lanes 2 and 3). Oct1 binding activities served as controls for the amount and quality of nuclear extracts. These results suggest that E protein functions can be manipulated by expression of Id1 and E47-ER as expected.

Next, we performed DNA microarray analyses with total RNA isolated from these cells and the GeneChip^® ^Mouse Genome 430 2.0 Array from Affymetrix (Santa Clara, CA) according to manufacturer's instructions. We set the criteria for genes of significance to be those whose expression is down regulated at least 2-fold by Id1 and reverted over 2-fold by E47-ER, or vice versa. Furthermore, the expression level of these genes must be at least 2-fold higher than background and receive a "present call" determined by the computer program designed by Affymetrix. Thus, we obtained 438 genes down-regulated by Id1 compared to vector-transduced cells. Out of these genes, 203 genes were activated by E47-ER after induction with 4-OHT for one hour. Table 1 lists the first 25 genes whose expression is most dramatically reduced by Id1. Topping this list is the RORγ gene, which was down-regulated 44-fold by Id1 and re-activated 22-fold by E47-ER. However, a large fraction of genes on this list have unknown function. Therefore, their relevance to T cell development was not immediately apparent. Despite this, several genes previously known to be regulated by E2A are among the group of 203 identified, which provided us with confidence in the procedure. For example, the Cdkn1a (p21) gene was down and up-regulated by about 5-fold, respectively, whereas expression of CD4 and RAG1 or 2 genes was altered by 2–3 fold [29-31].

**Table 1:** Genes regulated by Id1 and E47-ER

**Accession number**	**Fold ↓ by Id1**	**Fold ↑ by E47ER**	**Genes**	**Accession number**	**Fold ↑ by Id1**	**Fold ↓ by E47ER**	**Genes**
AF163668	44.22	21.82	RORc^#^	U43884	857.89	0.49	Idb1
BB391602	42.06	14.56	EST	BB703880	137.38	2.09	EST
AW492576	35.85	23.38	EST	X03802	91.66	0.54	
AK020348	33.65	34.20	Acbd7	BB199001	84.05	0.54	
BB204492	33.38	32.92	EST	BG070914	43.65	1.59	
BB702377	31.11	3.96	EST	NM_011395	42.49	1.56	
NM_008179	25.27	16.41	Gspt2	BF456582	38.25	2.22	EST
BE981638	24.18	14.79	EST	BC021939	36.72	2.78	osteoglycin
BM239905	23.17	25.83	EST	C79967	36.43	1.05	
BB045044	23.15	4.44	EST	AB013605	35.39	29.63	Per3
AV341285	22.55	5.38	EST	BB364262	34.29	1.55	
BB780056	21.67	17.51	EST	AV354897	33.43	17.12	EST
BI732921	19.50	8.13	EST	BB206037	32.75	1.58	
BG071830	18.46	1.37		BB283894	31.92	0.92	
BB748702	16.95	1.97		BB217381	31.18	1.91	
AK008128	16.87	2.11	Unknown*	AU080926	30.46	0.72	
NM_025422	16.78	7.84	CD302	BB023986	28.53	1.24	
BC021465	16.77	6.08	unknown	BB346879	27.46	0.97	
BG065821	15.67	6.42	EST	BB005315	27.24	0.90	
AV013697	15.56	8.41	EST	AK021082	25.85	7.48	unknown
BE956696	15.51	11.60	EST	AU022985	24.86	10.15	EST
BB375478	15.00	10.40	EST	BE649198	23.98	0.95	
BM245920	14.90	6.69	EST	AK017568	23.70	1.27	
BB366565	14.83	21.44	EST	NM_007518	23.48	1.86	
AV303905	14.53	14.12	EST	AF054581	22.37	1.39	

# also known as RORγ; * cDNA with unknown function.

In contrast, we found 1547 genes to be up-regulated by Id1, of which only 253 genes were repressed by E47-ER within 1 hour. Since Id1 is known to be an inhibitor of E2A transcription activators, it is reasonable that a smaller percentage of the up-regulated genes are direct consequences of loss of E protein function. Rather, expression of the majority of the genes may be indirectly influenced by cellular conditions altered by Id1 expression. For example, of the first 25 genes whose expression was up-regulated by Id1, only 7 of them were repressed by E47-ER (Table 1). This compared to 24 of the 25 genes, which were down-regulated by Id1 and re-activated by E47-ER. However, it will be interesting to further verify if the 253 genes are directly repressed by E2A. As expected, Id1 itself was found to be the gene expressed at the highest level (858 fold) but was not repressed by E47-ER, further validating our experimental procedures.

### RORγ is a direct target gene of E2A

**Figure 2:**
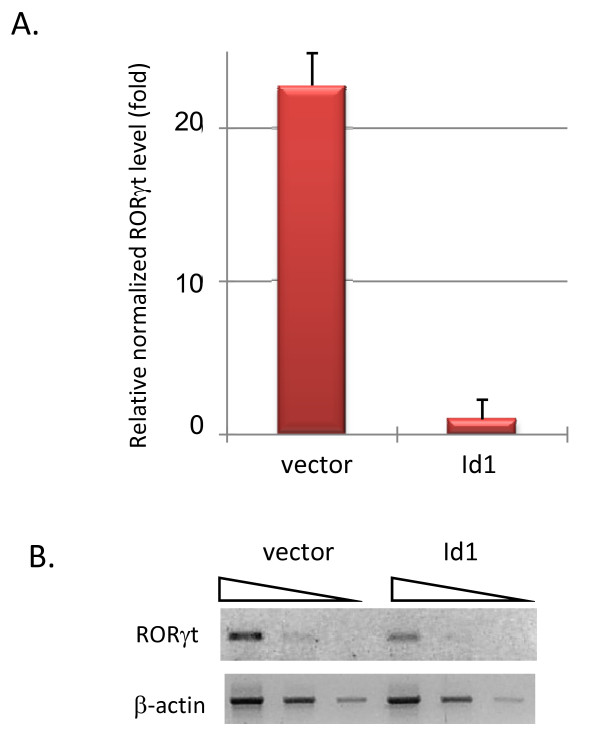
**Regulation of RORγt expression by Id1 and E47**. (A) Real-time reverse transcriptase-PCR assays were performed using RORγt or β-actin specific primer sets on cDNA samples from vector or Id1 transduced 16610D9 cells. The primer sequences for RORγt are GTGTGCTGTCCTGGGCTACC and AGCCCTTGCACCCCTCACAG. Those for β-actin are GGCTGTATTCCCCT CCATCG and CCAGTTGGTAACAATGCCATGT. The Ct values of RORγt were normalized against those of β-actin. Data are presented relative to the normalized RORγt level in Id1 expressing cells, with standard deviations obtained from at least three data points for each sample. (B) RT-PCR assays were conducted on serially diluted cDNA samples of primary thymocytes transduced with vector or Id1-expressing retroviruses.

The RORγ gene encodes two transcripts, RORγ and RORγt [23,32]. RORγt is specifically expressed in T cells from an internal promoter located between exons 1 and 2 of the gene. Using primers specific for RORγ and RORγt transcripts, we found by RT-PCR that RORγt but not RORγ was expressed in 16610D9 cells. Real-time PCR assays confirmed that RORγt expression was diminished in Id1 expressing cells by 23 fold, compared to cells transduced with vector control (Fig. 2A). To test if Id1 expression in primary thymocytes inhibits RORγt expression, we transduced wild-type thymocytes with vector or Id1-expressing retroviruses, sorted transduced cells based on GFP expression and measured RORγt levels using RT-PCR. Indeed, RORγt levels were significantly reduced by Id1 (Fig. 2B). Together, these results confirmed that RORγt expression could be inhibited by Id1.

**Figure 3:**
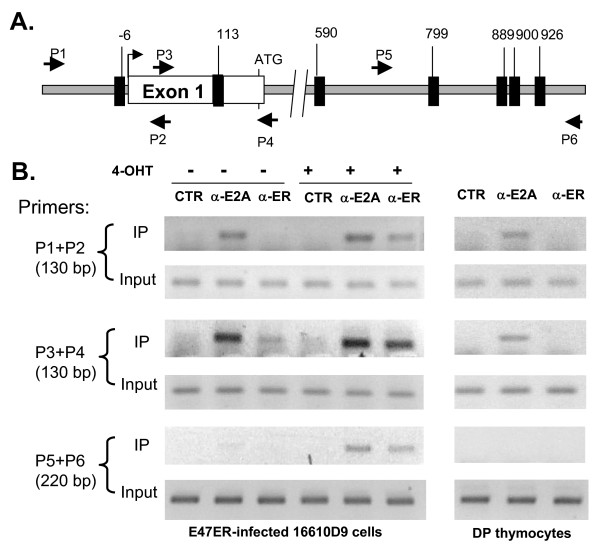
**E2A binds near the RORγt promoter region**. (A) Schematic representation of the RORγt gene. Exon1 is shown as a white box with transcription start site marked by an arrow and the ATG codon labeled. Dark boxes represent E boxes (RCAGNTG) and their location is denoted by the number of nucleotides relative to the transcription start site. Small arrows signify primers used for PCR. (B) Chromatin immunoprecipitation assays with indicated antibodies and primer sets. Control (CTR) antibodies used were anti-CD3 antibodies. E47-ER transduced 16610D9 cells treated with or without 4-OHT and DP thymocytes were used in the assays. PCR performed with 2% of total input DNA served as controls.

To further verify if RORγt is indeed a target gene of E proteins, we tested if E2A is bound to the promoter region of the RORγt using chromatin immunoprecipitation assays (Fig. 3). Several E boxes, to which E proteins bind, were found near the RORγt promoter region (Fig. 3A). We used E47-ER expressing 16610D9 cells or primary DP thymocytes for these assays. PCR products containing the two proximal E boxes were detected (by amplifying with primer pairs 1–2 and 3–4) in the anti-E2A immunoprecipitates from 16610D9 cells treated with or without 4-OHT (Fig. 3B). In contrast, anti-ER antibodies only brought down these fragments from 16610D9 cells induced with 4-OHT, which demonstrated the specificity of the assay. Furthermore, control antibodies against an irrelevant protein did not precipitate any DNA in this region. The distal E boxes included in the fragment amplified by primers 5 and 6 were only brought down when E47-ER was induced, suggesting that these E boxes are unlikely to be bound with physiological levels of E proteins. From primary DP thymocytes, anti-E2A but not anti-ER antibodies pulled down DNA containing the two proximal E boxes but not the distal ones, demonstrating the specific binding by endogenous E2A proteins (Fig. 3B). Collectively, these data suggest that RORγt is likely a direct target of E proteins in vivo.

### Effects of RORγt on Id1-induced apoptosis

**Figure 4:**
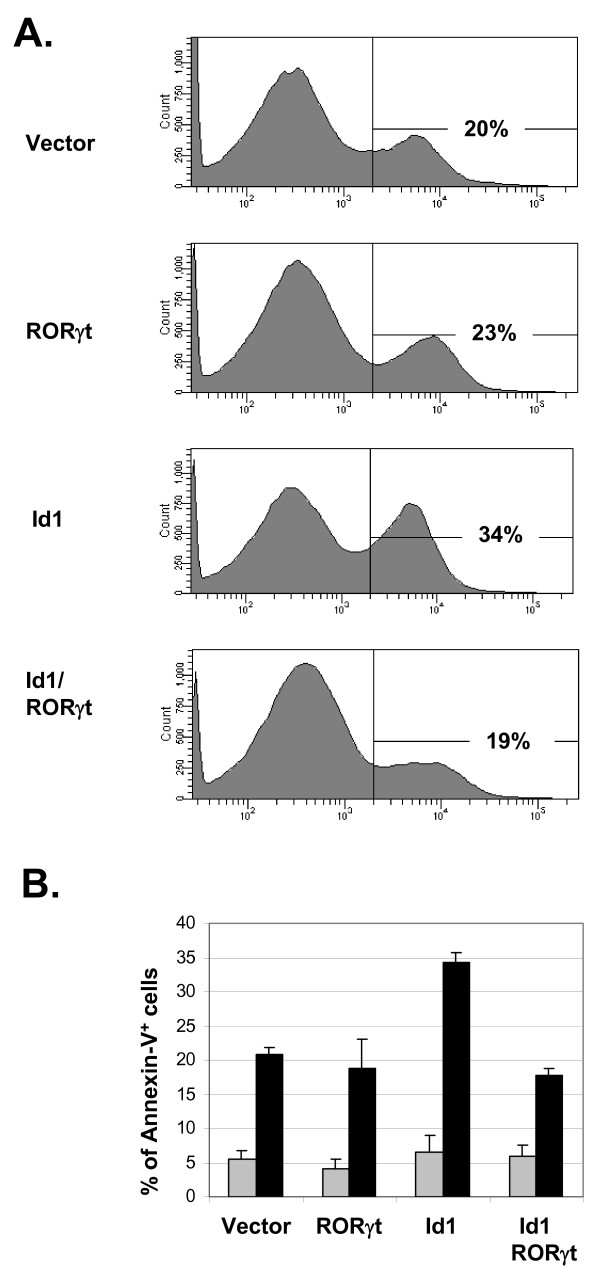
**RORγt rescues Id1-induced apoptosis**. (A) Annexin V staining. 16610D9 cells were transduced with indicated retroviruses and sorted based on GFP or YFP expression from the RORγt and Id1 retrovectors, respectively. Sorted cells were cultured for 6 days before the analysis. Cells negative for 7-AAD staining were scored for Annexin-V binding by percentage. (B) Average percentages of Annexin-V positive cells were obtained from three independent experiments. Data obtained on Day 6 are shown in dark bars and those on Day 1 are presented in gray and serve as negative controls. Error bar shows standard deviation.

It has previously been shown that Id1 expression causes massive apoptosis of developing thymocytes [18,20]. Because RORγt has been implicated in protection of double positive thymocytes from apoptosis, we examined the effect of RORγt on Id1-induced apoptosis of 16610D9 cells [25]. We transduced the cells with retroviruses expressing Id1 or RORγt individually or together. Transduced cells were sorted based on GFP or YFP expression and cultured for 6 days. Cells were then stained with PE-conjugated Annexin-V and 7AAD. Annexin-V positive and 7AAD negative cells were considered apoptotic cells. Expression of Id1 in 16610D9 cells resulted in about 34% Annexin-V positive cells, whereas the percentages in vector or RORγt transduced cells were only about 20% (Fig. 4A). Co-expression of Id1 with RORγt reduced the percentage of apoptotic cells back down to 19%. These experiments were repeated multiple times and the averages of Annexin-V positive cells are shown in Fig. 4B. As controls, we stained cells cultured one day after sorting and found no significant difference in percentages of apoptotic cells (Fig. 4B). These results suggest that reduction in RORγt expression is a potential cause for apoptosis occurring in Id1-expressing cells.

### RORγt selectively prevents Id1-induced p38 MAP kinase hyper-activation

**Figure 5:**
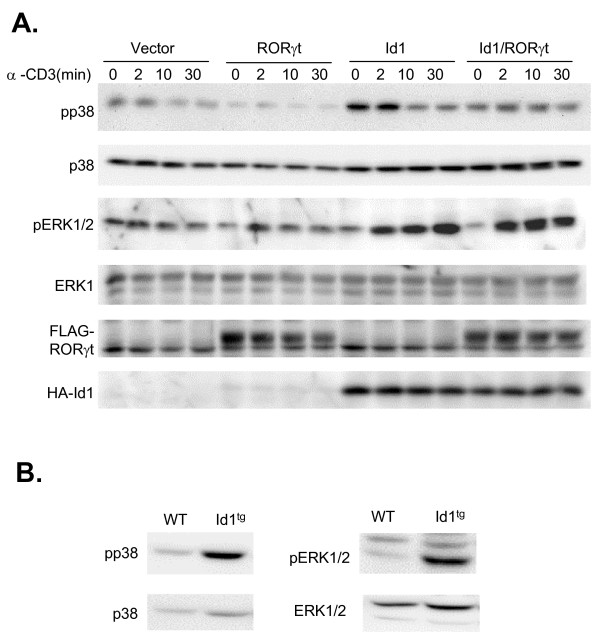
**RORγt prevents Id1-induced p38 hyper-activation**. (A) Immunoblot analyses of MAP kinase activation in the absence or presence of TCR signaling. 16610D9 cells were transduced with indicated retroviruses and stimulated with soluble anti-CD3 antibodies plus protein A-streptavidin for indicated lengths of time. Whole cell lysates were used for immunoblotting with antibodies against indicated proteins. (B) Immunoblot analysis of MAP kinase activation in sorted DP thymocytes.

p38 MAP kinase is often activated in cells under stress or undergoing apoptosis [33,34]. Therefore, we measured p38 activation in Id1 and/or RORγt expressing 16610D9 cells. Since T cell receptor (TCR) signaling is known to influence MAP kinase activities [35], we also examined MAP kinase activation following TCR signaling triggered by incubation with anti-CD3 antibodies. Even in the absence of anti-CD3, the level of phospo-p38 (pp38) was dramatically increased by Id1 but not by RORγt. RORγt appeared to decrease the level of pp38 compared to vector control but this became insignificant when normalized against total amounts of p38 in each lane. However, co-expression of Id1 with RORγt abolished the positive effect of Id1 on p38 activation before and after anti-CD3 stimulation (Fig. 5A). This effect of RORγt is consistent with its anti-apoptotic function shown in Fig. 4. However, whether p38 activation is directly regulated by RORγt or indirectly influenced by the overall cellular condition remains to be investigated.

In contrast to p38 activation, Erk1 and 2 were phosphorylated immediately following TCR signaling. TCR-induced activation of Erk1 and 2 was potentiated by Id1 expression, although basal levels of Erk1 and 2 present in the absence of TCR stimulation were not affected. However, RORγt had no effect on Erk activation in the absence or presence of Id1 (Fig. 5A). Therefore, these results provided supporting evidence that RORγt specifically counteracts Id1-induced p38 activation. Id1 expression did not alter JNK activities with or without TCR stimulation (data not shown). Therefore, the effect of RORγt on JNK was not examined.

Consistent with our findings in 16610D9 T cells, we have also detected elevated levels of phospho-p38 in double positive thymocytes freshly isolated from Id1 transgenic mice (Fig. 5B). Likewise, levels of activated Erk were also increased in these cells. Thus, these data indicate that Id1 expression alters MAP kinase signaling not only in T cell lines but also in thymocytes of transgenic mice.

## Discussion

We have presented data to suggest that the RORγ gene is a direct target of E2A transcription factors, whose function can be inhibited by Id1 and loss of RORγt expression could, at least in part, account for the pro-apoptotic effect of Id1. These data are consistent with those noted by others including the finding that thymocytes of transgenic mice expressing SCL/Tal1, another inhibitor of E proteins, have reduced expression of RORγ [26,36]. Expression of Id1 in transgenic mice not only blocks T cell differentiation but also causes massive apoptosis [18]. Examination of the apoptotic cells reveals that these cells have functionally rearranged TCRβ genes and are likely double positive thymocytes [20]. Interestingly, RORγ deficient mice also display similar phenotypes characterized by reduced numbers of DP thymocytes and massive apoptosis in the thymus [24,25]. These similarities, together with the fact that RORγt rescues Id1-induced cell death in 16610D9 cells, lend support to the notion that reduction in RORγt expression due to inhibition of E protein function may contribute to apoptosis of developing thymocytes in Id1 transgenic thymuses. However, this may not be the only reason, because the apoptotic phenotype of RORγ deficient mice can be rescued by transgenic expression of Bcl-xL [25] whereas expression of Bcl2 does not protect Id1 transgenic thymocytes (data not shown). Although Bcl-xL is thought to be a target of RORγt [25], we could not evaluate the effect of Id1 on its expression because 16610D9 cells produce very low levels of Bcl-xL.

RORγt has also been shown to be able to suppress cell proliferation [26,37]. Because precursor T cells undergo tremendous expansion during their differentiation, levels of RORγt must be carefully modulated to maintain a balance between proliferation and survival. Interestingly, the function of E proteins, which are known to inhibit cell cycle [8], is also temporally controlled in this process. Namely, Id3 gene expression is transiently activated following pre-TCR signaling via the Ras-MAP kinase pathway, leading to inhibition of DNA binding by E proteins [15]. Consequently, loss of E protein function may result in reduced RORγt expression, thus allowing cell proliferation to proceed. When Id3 expression decreases and E protein function recovers, RORγt expression returns to suppress cell cycle progression and facilitate the survival of DP thymocytes. Because of the growth suppressive function of RORγt, its overexpression under the CD2 promoter resulted in dramatically reduced cellularity in transgenic mice [37]. The CD4 promoter drives RORγt expression in a fraction of DP thymocytes of transgenic mice [38]. However, when these mice were crossed with Id1 transgenic mice, RORγt-expressing cells as indicated by the fluorescence of GFP co-expressed from the same transgenic construct were drastically under-represented (Yang and Sun, data not shown). It is not clear if this is due to inhibition of the CD4 promoter by Id1 or selective elimination of RORγt expressing cells by Id1. Nevertheless, these complications prevented us from determining if RORγt could rescue cell death and influence T cell development or tumorigenesis in animals.

We have shown that both Erk and p38 MAP kinases are hyper-activated in DP cells of Id1 transgenic mice, which may have profound effects on developing thymocytes, leading to their death. It is interesting that RORγt expression in 16610D9 cells could prevent Id1-induced hyper-activation of the p38 MAP kinase. This may be an indication of the protective effect of RORγt on Id1-expressing 16610D9 cells since we have found that apoptosis as measured by Annexin-V staining is alleviated by RORγt. Alternatively, p38 activation may play an active role in triggering cell stress responses and apoptosis. RORγt may act by inhibiting p38 activation and protecting cells from apoptosis. In contrast to p38, Erk hyper-activation following TCR signaling is not suppressed by RORγt, which suggests that Id1 affects Erk and p38 through different mechanisms. It is possible that Erk activation depends on TCR signaling and Id1 expression influences the signaling pathway through an unknown mode of action. This is consistent with our previous findings that Id1 lowers the threshold of pre-TCR or TCR signaling and alters the outcome of T cell selection processes [19,20]. On the other hand, Id1 expression may also affect cell survival processes that are downstream or independent of TCR signaling by inhibiting RORγt expression. The fact that Id1 is specifically turned on during negative but not positive selection suggests that Id1 might play a role in eliminating self-reacting thymocytes [17]. Taken together, these observations illustrate the complexity of coordinated molecular events that ensure proper development of T lymphocytes.

E proteins act as important regulators of T cell differentiation by controlling multiple crucial checkpoints in the process, perhaps by regulating transcription of many different genes. Our microarray analyses revealed RORγt as a strong candidate to be positively controlled by E proteins. Several additional candidates scored comparably to RORγt but their functions are unknown or their connections with T cell biology are not apparent. Future investigation might uncover novel links between these other genes controlled by E proteins and provide a more complete description of the function of E proteins in T cell development.

## Conclusion

This study highlights the link between E protein controlled RORγt expression and apoptosis of Id1-expressing T cells. Because Id1 is a natural inhibitor of E proteins and is specifically turned on during negative selection of T cells, findings described here will likely be of physiological significance and shed light on mechanisms regulating T cell maturation.

## Methods

### Cell culture and retroviral transduction

The retroviral construct expressing Id1 is as described except that EGFP was replaced with YFP [27]. To express E47-ER, an EcoRI fragment containing the coding sequence of human E47 without the stop codon was fused with another EcoRI fragment encoding the modified hormone-binding domain of estrogen receptor, which preferentially binds tamoxifen [28]. Both EcoRI fragments were cloned into the EcoRI site of the MIGR1 vector that also expresses EGFP from an internal ribosome entry site [39]. For the RORγt expression vector, a BamHI-EcoRI fragment generated by RT-PCR with 5' (GGATCCatgagaacacaaattg aagtgatc) and 3' (GAATCCTCACTTTGACAGCCCCTCAGG) primers was cloned into Bgl II-EcoRI site of MIGR1 with a HPC4 tag at the 5' end.

Retroviruses were produced by transient transfection of the Phoenix-E packaging cell line [40]. Culture supernatants were collected and spin-infection of 16610D9 cells was carried out by centrifuging the cells in viral supernatants for two hours in the presence of 4 μg/ml polybrene followed by incubation at 37°C for an additional two hours. Cells were then cultured in fresh medium at 37°C for 20 hours before sorting. To transduce primary thymocytes, thymocytes isolated from wild type mice were mixed with viral supernatants containing 4 μg/ml polybrene and incubated at 25°C for 30 min before spin-infection. Transduced cells were sorted using Moflo (Dako, Denmark) with the capability to separate GFP and YFP-expressing cells. Sorted GFP and/or YFP positive cells were cultured overnight at 37°C before treatment with 1 μM 4-OH-tamoxifen.

### Microarray analysis

Total RNA was extracted using TRIzol reagent (Invitrogen) and further purified with RNeasy kit (Qiagen, Valencia, CA). Target probes for microarray analysis were prepared as described in the Affymetrix GeneChip Expression Analysis Manual. Briefly, 5 μg of total RNA was reverse transcribed and double-stranded cDNAs were synthesized with the Superscript II cDNA System (Invitrogen, Carlsbad, CA) using T7-Oligo(dT)24 primer. Double-stranded cDNA was purified using the GeneChip Samples Cleanup Module (Affymetrix, Santa Clara, CA) and used for synthesis of biotin-labeled antisense cRNA with BioArray High Yield RNA Transcript Labeling IVT Kit (Affymetrix). cRNA was purified using GeneChip Sample Cleanup Module for IVT cRNA (Affymetrix). Twenty μg of cRNA was fragmented in 1× fragmentation buffer (40 mM Tris-acetate (pH 8.1), 100 mM KOAc, 30 mM MgOAc) for 35 min at 94°C. Eighteen μg of the fragmented cRNA was used to hybridize with Affymetrix Genechip Mouse Genome 430 2.0 chips for 16 h at 45°C in an Affymetrix Hybridization Oven 640 while rotating at a speed of 60 rpm. The chips were washed and stained with R-Phycoerythrin Streptavidin in Affymetrix Fluidics Station 450 according to an Affymetrix GeneChip protocol and scanned using an Affymetrix GeneChip Scanner 3000. The preliminary data were analyzed using Affymetrix Microarray Suite 5.0 and exported to Microsoft Excel for further analysis.

### Chromatin immunoprecipitation assays

ChIP assays were performed using ChIP assay kit (Millipore, Billerica, MA) according to manufacturer's instructions. 1 × 10^7 ^cells were fixed and lysed in lysis buffer in the presence of protease inhibitors. Lysate was sonicated to shear DNA into 300–500 bp fragments and immunoprecipitated with antibodies against E47 or estrogen receptor (ER) (Santa Cruz Biotechnology, Santa Cruz, CA) as well as CD3 (BD Pharmingen, San Diego, CA). Immunoprecipitated DNA was recovered by proteinase K digestion and used for PCR amplification for 30 or 33 cycles along with input DNA (2%) as controls. PCR products were resolved using 2% agarose gel. Primers used for PCR are P1, GTGCCTGTCATCATACC CAATGC; P2, gtgcctcagcccttagatctgcac; P3 GTGCAGATCTA AGGGCTGAGGC; P4 CATTCACTTACTTCTCATGACTG; p5 GACTGCTC TGTGTGTGCTATGTG and p6 GCTTCCCTTACATGAGCAGGGAGGATC.

### Immunoblotting

Sorted primary thymocytes or 16610D9 cells with or without stimulation were lysed in radioimmuno-precipitation buffer (137 mM NaCl, 2.7 mM KCl, 4.3 mM Na_2_HPO4, 1.4 mM KH_2_PO4, 1% Nonidet P-40, 0.5% sodium deoxycholate, and 0.1% SDS) plus cocktails of protease and phosphatase inhibitors, and supernatants were collected after centrifugation. Protein concentrations were determined by using the BCA reagents (Pierce, Rockford, IL). Thirty micrograms of each protein extract was analyzed using SDS-polyacrylamide gels. Antibodies specific for p38, phospho-p38, ERK1, and phospho-ERK1/2 were purchased from Cell Signaling Technology (Beverly, MA); anti-Flag and anti-HA antibodies were from Sigma (St. Louis, MO).

### Apoptosis assay

Retrovirally transduced cells were sorted and cultured for 6 days. Cells were then washed with 2× PBS and stained with Annexin V-PE together with 7-AAD (7-Amino-Actinomycin D) for 15 minutes at room temperature in the dark as suggested by the manufacturer (BD Pharmingen). Stained Cells were analyzed using flow cytometry on LSR II (BD Pharmingen), apoptotic cells were defined as Annexin-PE positive and 7-AAD negative.

## Abbreviations

DN: double negative; DP: double positive; SP: single positive; RORγ: retinoid-related orphan receptor gamma; Id: inhibitor of differentiation

## Authors' contributions

YY designed and conducted experiments and wrote part of the manuscript. HCW conducted experiments. XHS designed experiments and wrote the manuscript.
